# Specialty Palliative Care and Symptom Severity and Control in Adolescents and Young Adults With Cancer

**DOI:** 10.1001/jamanetworkopen.2023.38699

**Published:** 2023-10-20

**Authors:** Sumit Gupta, Qing Li, Alisha Kassam, Adam Rapoport, Kimberley Widger, Karine Chalifour, Nancy N. Baxter, Paul C. Nathan, Natalie G. Coburn, Rinku Sutradhar

**Affiliations:** 1 Division of Haematology/Oncology, The Hospital for Sick Children, Toronto, Ontario, Canada; 2 Faculty of Medicine, University of Toronto, Toronto, Ontario, Canada; 3 Cancer Research Program, ICES (Institute for Clinical Evaluative Sciences), Toronto, Ontario, Canada; 4 Institute for Health Policy, Evaluation and Management, University of Toronto, Toronto, Ontario, Canada; 5 Department of Pediatrics, Southlake Regional Health Centre, Newmarket, Ontario, Canada; 6 Division of Paediatrics, Faculty of Medicine, University of Toronto, Toronto, Ontario, Canada; 7 Division of Family and Community Medicine, Faculty of Medicine, University of Toronto, Toronto, Ontario, Canada; 8 Emily’s House Children’s Hospice, Toronto, Ontario, Canada; 9 Paediatric Advanced Care Team, The Hospital for Sick Children, Toronto, Ontario, Canada; 10 Lawrence S. Bloomberg Faculty of Nursing, University of Toronto, Toronto, Ontario, Canada; 11 Young Adult Cancer Canada, St John’s, Newfoundland and Labrador, Canada; 12 Li Ka Shing Knowledge Institute, St Michael’s Hospital, Toronto, Ontario, Canada; 13 Melbourne School of Population and Global Health, University of Melbourne, Melbourne, Victoria, Australia; 14 Department of Surgery, Sunnybrook Health Sciences Centre, Toronto, Ontario, Canada; 15 Department of Surgery, University of Toronto, Toronto, Ontario, Canada; 16 Sunnybrook Research Institute, Toronto, Ontario, Canada; 17 Dalla Lana School of Public Health, University of Toronto, Toronto, Ontario, Canada

## Abstract

**Importance:**

Adolescents and young adults (AYAs) with cancer experience substantial symptom burden. Specialty palliative care (SPC) is recommended but often not involved or involved late.

**Objectives:**

To determine whether patient-reported symptom severity was associated with subsequent SPC involvement and whether SPC was associated with symptom improvement in AYAs with cancer.

**Design, Setting, and Participants:**

This cohort study comprised AYAs (aged 15-29 years) with primary cancer diagnosed between January 1, 2010, and June 30, 2018, in Ontario, Canada. Data, including self-reported Edmonton Symptom Assessment System (ESAS) scores, were obtained from health care databases. Specialty palliative care was identified through billing codes and validated algorithms. Final data analysis was performed on April 4, 2023.

**Main Outcomes and Measures:**

Associations of ESAS scores with subsequent SPC involvement were determined. A difference-in-differences approach was used for patients who died within 5 years of their cancer diagnosis. Case patients (SPC predeath, index date equals first SPC service) were matched 1:1 to control patients (no SPC at equivalent time before death). The study examined whether the difference between 90-day postindex and preindex mean ESAS scores was itself different between case and control patients.

**Results:**

This study included 5435 AYAs with cancer, with a median follow-up of 5.1 (IQR, 2.5-7.9) years for analyses of general palliative care. Their median age at cancer diagnosis was 25 (IQR, 22-27) years, and more than half were male (2809 [51.7%]). For all symptoms, moderate and severe ESAS scores were associated with an increased likelihood of SPC involvement compared with mild scores. The greatest magnitude of association was seen for pain scores (hazard ratio for severe vs mild, 7.7 [95% CI, 5.8-10.2]; *P* < .001). A total of 721 AYAs (13.3%) died within 5 years of diagnosis, and 612 of these patients (84.9%) had received SPC before death. Among 202 case-control pairs, SPC involvement was associated with improved pain trajectories (mean scores improved from 3.41 to 3.07 in case patients and worsened from 1.86 to 2.16 in control patients; *P* = .003). Other symptom trajectories were not affected.

**Conclusions and Relevance:**

In this cohort study of AYAs with cancer, those reporting moderate or severe symptoms through a screening program were more likely to subsequently receive SPC. These findings suggest that SPC was associated with a subsequent decrease in pain severity but did not affect other symptoms. New interventions targeting other symptoms during treatment and particularly at the end of life are needed.

## Key Points

**Question:**

Is specialty palliative care (SPC) associated with improvement in symptoms among adolescents and youg adults (AYAs) with cancer?

**Findings:**

In this cohort study of 5435 AYAs with cancer, increased symptom severity was associated with subsequent SPC involvement. Specialty palliative care was associated with improvement in pain but did not affect other symptoms.

**Meaning:**

These findings suggest that further research identifying effective interventions for nonpain symptoms in AYAs is required, particularly at the end of life.

## Teaser

This cohort study examines the association between specialty palliative care and symptom improvement among adolescents and young adults with cancer.

## Introduction

Adolescents and young adults (AYAs) with cancer are a vulnerable subgroup at risk of inferior outcomes.^1,2,3^ Adolescents and young adults also experience substantial symptom burden that differs from that of younger and older patients.^4,5,6,7,8^ Palliative care (PC) is an essential part of cancer care that should be integrated into treatment early in the disease course.^9^ Multiple studies have highlighted unique challenges in delivering PC to AYAs, given different developmental and psychosocial needs.^10,11,12^ Even among AYAs with cancer who die, PC is often not involved or involved late despite observed benefits such as a decreased likelihood of high-intensity care.^13,14^ Increasing the uptake of PC among AYAs with cancer is crucial, but how to do so is unclear. In addition, evidence supporting the efficacy of PC in improving symptoms is scarce but would inform efforts to improve access to PC in this population.

We therefore leveraged population-based databases to identify a cohort of AYAs with cancer and their patient-reported symptom burden. Our primary objectives were (1) to determine whether symptom severity was associated with subsequent PC involvement and (2) to determine whether PC involvement was associated with subsequent symptom improvement.

## Methods

Ethics approval was obtained from The Hospital for Sick Children and Sunnybrook Health Sciences Centre. Informed consent was not required given the use of only deidentified data, strict privacy regulations to address any risk of reidentification, and the lack of feasibility to contact patients. The study followed the Strengthening the Reporting of Observational Studies in Epidemiology (STROBE) reporting guideline.

### Study Setting

Canadian health care is delivered by provincial governments through universal health insurance plans. In Ontario, cancer care delivery is overseen by Ontario Health. Adult cancer care is delivered through both regional cancer centers (RCCs) and community hospitals. Pediatric institutions do not generally treat patients aged 18 years or older. No organized provincial AYA cancer programs or facilities exist.

### Symptom Scores

The Edmonton Symptom Assessment System (ESAS) is a validated patient-reported measure that assesses the presence and severity of 9 cancer-associated symptoms: pain, tiredness, drowsiness, nausea, lack of appetite, shortness of breath (dyspnea), depression, anxiety, and overall well-being.^15,16^ Each symptom is scored on an 11-point numeric scale from 0 (no symptoms) to 10 (worst possible symptoms), commonly categorized as no symptoms (0), mild (1-3), moderate (4-6), and severe (7-10).^17,18^ In 2007, Ontario Health implemented a provincial program that aimed to screen patients at cancer-related outpatient visits using the ESAS to optimize symptom control. The ESAS screening was broadly available at RCCs by 2010.^19,20,21^ Implementation among non-RCCs is more variable. Inpatients are not screened. Symptom scores are collected centrally in the Symptom Management Reporting Database.^19,20,21,22^

### Association of Symptom Severity With Subsequent PC Involvement

#### Study Population

We created a retrospective population-based cohort of AYAs who were aged 15 to 29 years at primary cancer diagnosis between January 1, 2010, and June 30, 2018, as identified through the Ontario Cancer Registry.^22^ Populations who did not have ESAS access were excluded if they (1) received treatment in pediatric institutions, (2) did not require services at a RCC in the first year after diagnosis (eg, surgically resected thyroid cancer), or (3) were treated exclusively at non-RCCs. Using unique encoded identifiers, patients were linked to population-based health services databases housed at ICES (formerly the Institute for Clinical Evaluative Sciences), a research institute encompassing an array of Ontario health-related data (eTable 1 in Supplement 1). Race and ethnicity are not routinely collected in Ontario health-related data.

#### Outcomes

The primary outcomes were time to first general PC (GPC) or specialty PC (SPC) visit. Palliative care visits were defined using validated algorithms of PC-related billing codes (eAppendix 1 in Supplement 1). Specialty PC visits were defined as any PC billing code submitted by physicians for whom PC claims comprised more than 10% of their previous year’s total.^23^ This cutoff was previously determined to best identify Ontario physicians who self-declared as practicing mostly PC.^23^ All other visits were defined as GPC, representing generalist-level PC provided by family physicians, oncologists, or internists.^23^

#### Key Variable and Covariates

The ESAS score was conceptualized as a time-varying variable: not measured vs none or mild (score 0-3, hereinafter referred to as mild) vs moderate (4-6) vs severe (7-9). If more than 28 days passed after an ESAS score without a subsequent measurement, patients were recategorized as “not measured.” Each ESAS symptom was considered separately.

Covariates included age at diagnosis (continuous) and sex. Neighborhood income quintile and urban or rural status were determined using Canadian census data.^24,25^ Regional location was categorized as 1 of the 5 main Ontario health regions (Central, East, North, Toronto, or West). Cancer type was categorized as hematologic, melanoma, central nervous system, sarcoma, testicular or ovarian, breast, colorectal, thyroid, or other. The time period of diagnosis was defined as early (2010-2014) or late (2015-2018).

### Association of PC Involvement With Subsequent Symptom Improvement

**Figure 1.**
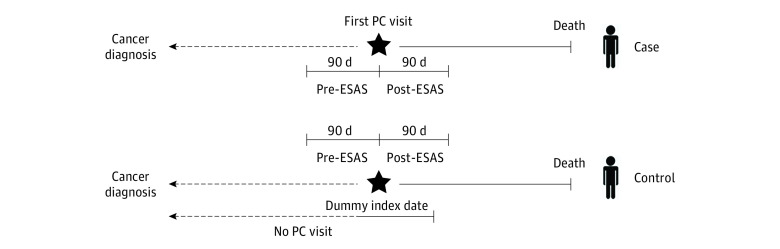
Case-Control Design Among the Decedent Cohort ESAS indicates Edmonton Symptom Assessment System; PC, palliative care.

The second set of analyses was restricted to patients who died within 5 years of cancer diagnosis. We used a matched difference-in-differences study design.^26^ Two sets of case patients were defined based on (1) GPC involvement and (2) SPC involvement. For GPC, case patients comprised decedent patients with GPC involvement between cancer diagnosis and death, and with least 1 ESAS measurement in both the 90 days before and the 90 days after initial GPC involvement (ie, the index date; Figure 1). The ESAS measurements on the date of initial GPC service were considered before GPC involvement. For each case patient, all possible decedent control patients were then identified based on the same index date, defined by the time before death (eg, if the case patient’s first GPC involvement occurred 6 months before death, the control patient’s dummy index date was also defined as 6 months before death). Control patients must also have had at least 1 ESAS measurement in the 90 days before and after their dummy index date. Control patients could not have had any GPC involvement during or before this window. Control patients were matched by sex and cancer type (hematologic vs solid tumor vs central nervous system) using a 1:1 matching ratio. Individual patients may have served as control patients to multiple case patients. An individual case patient may also have served as a control patient for a different case (eg, a patient who experienced late GPC involvement could have been a control for patients with earlier GPC involvement).

Case and control patients were then redefined using the same methodology. This time, however, we used SPC involvement to distinguish them and used time of first SPC involvement as the index date.

### Statistical Analysis

#### Association of Symptom Severity With Subsequent PC Involvement

The primary analysis examined the association between ESAS score and time to first GPC and time to first SPC. Each outcome was analyzed separately. The observation window started at the time of cancer diagnosis (index date). Patients were censored at death, emigration from Ontario (2 consecutive quarters of ineligibility for the Ontario Health Insurance Program), or December 31, 2020, whichever came first. The cumulative incidence function estimated the probability of receiving each outcome over time. Cox proportional hazards regression, a time-to-event regression model, estimated the association between symptom severity and each outcome, with ESAS score incorporated as a time-varying measure. Sensitivity analyses limited the observation window to either 1 or 3 years. Unadjusted associations between ESAS score and time to PC visit were determined and then adjusted for all covariates.

#### Association of PC Involvement With Subsequent Symptom Improvement

For each case patient and each control patient, all ESAS scores in the 90 days before and after the index date were averaged to calculate mean preindex and postindex ESAS scores. The primary analysis determined whether the difference between a patient’s postindex and preindex mean ESAS scores was itself different between case patients (who had received GPC or SPC at the index date) and control patients (who had not). This difference-in-differences approach models the set of all mean ESAS scores using multivariable linear regression and 3 independent estimator variables: whether the mean score belonged to a case or control patient, whether it represented a preindex score or postindex or dummy index score, and an interaction term between the 2. A statistically significant interaction term indicated that the difference between preindex and postindex symptom scores itself differed between case and control patients.

Statistical significance was defined as *P* < .05. Final analyses were performed on April 4, 2023, using SAS Enterprise Guide, version 7.15 (SAS Institute Inc).

## Results

### Association of Symptom Severity With Subsequent PC Involvement

Of the 9399 patients identified, 5435 AYAs met the inclusion criteria (eAppendix 2 in Supplement 1). Their median age at cancer diagnosis was 25 (IQR, 22-27) years; 2809 (51.7%) were male and 2626 (48.3%) were female.^22^ Cohort characteristics are presented in Table 1. The median follow-up was 5.1 (IQR, 2.5-7.9) years for analyses of GPC and 5.6 (IQR, 3.1-8.2) years for analyses of SPC. Among patients with at least 1 ESAS measurement, the median number of ESAS measurements was 7 (IQR, 3-14).

**Table 1.** Demographic and Disease Characteristics of the Study Cohort

Characteristic	Participants (N = 5435)^a^
Age, median (IQR), y	25 (22-27)
Sex	
Male	2809 (51.7)
Female	2626 (48.3)
Time period	
Early (2010-2014)	3260 (60.0)
Late (2015-2018)	2175 (40.0)
Neighborhood income quintile	
Rural	535 (9.9)
Urban	
1 (Lowest)	924 (17.0)
2	1026 (18.9)
3	952 (17.6)
4	975 (18.0)
5 (Highest)	1009 (18.6)
Cancer type	
Hematologic	1748 (32.2)
Melanoma	343 (6.3)
Central nervous system	335 (6.2)
Sarcoma	245 (4.5)
Testicular or ovarian	1159 (21.3)
Breast	361 (6.6)
Colorectal	197 (3.6)
Thyroid	331 (6.1)
Other	716 (13.2)
Ontario region	
Central	1737 (32.0)
East	1268 (23.3)
North	350 (6.4)
Toronto	585 (10.8)
West	1493 (27.5)

^a^ Unless specified otherwise, values are presented as No. (%) of patients.

**Figure 2.**
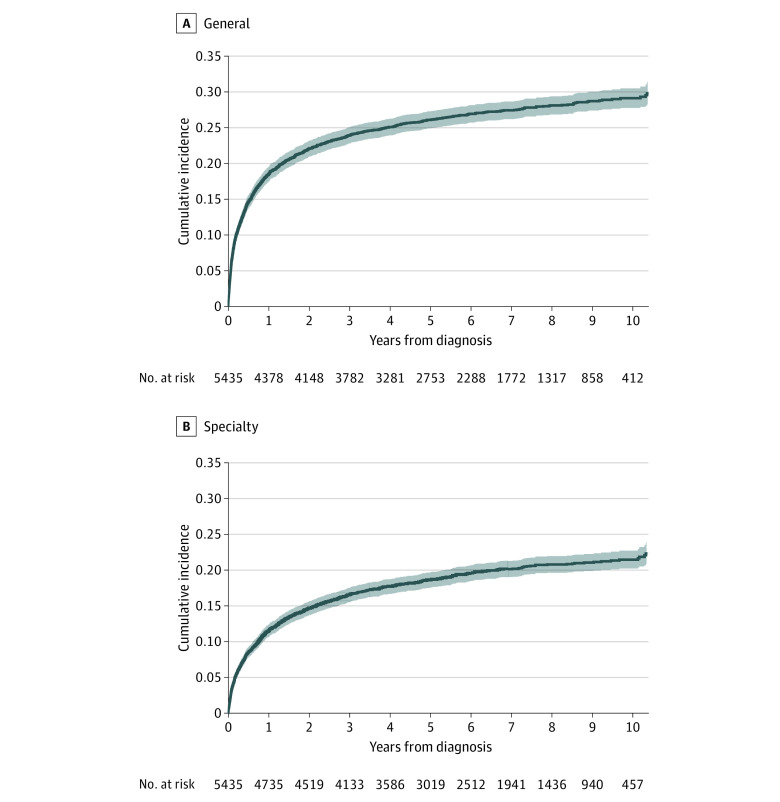
Cumulative Incidence of General and Specialty Palliative Care Involvement Blue lines represent cumulative incidence; shaded areas represent 95% CIs.

The 5-year cumulative incidence of GPC and SPC involvement was 26% (95% CI, 25%-27%) and 19% (95% CI, 18%-20%), respectively (Figure 2). Unadjusted and adjusted associations between ESAS scores and both GPC and SPC involvement are presented in Table 2. Compared with mild ESAS scores, moderate and severe scores were associated with increasing likelihood of both GPC and SPC involvement. Not being screened was associated with decreased likelihood. This pattern was consistent across symptom type, although the greatest magnitudes of association were seen in pain (adjusted hazard ratio [AHR] of SPC involvement for severe vs mild, 7.7 [95% CI, 5.8-10.2]; *P* < .001) and dyspnea (AHR, 5.4 [95% CI, 3.7-8.1; *P* < .001). The hazard ratios were consistently greater for SPC involvement vs GPC involvement. Adjustment for covariates resulted in minimal change in magnitudes of association compared with unadjusted results. Sensitivity analyses restricting the observation window to the first 3 years or first year after the initial diagnosis did not substantively change the results.

**Table 2.** Unadjusted and Adjusted Associations (HR Estimates) Between Edmonton Symptom Assessment System Scores and General and Specialty Palliative Care Involvement for Each Symptom

Symptom^a^	General palliative care, HR (95% CI)				Specialty palliative care, HR (95% CI)			
	Not measured	Mild	Moderate	Severe	Not measured	Mild	Moderate	Severe
Anxiety								
Unadjusted	0.6 (0.5-0.6)	1 [Reference]	1.6 (1.3-2.1)	2.2 (1.6-2.9)	0.4 (0.4-0.5)	1 [Reference]	1.9 (1.5-2.5)	2.6 (1.9-3.5)
Adjusted	0.6 (0.5-0.7)	1 [Reference]	1.6 (1.2-2.0)	2.0 (1.5-2.6)	0.5 (0.4-0.5)	1 [Reference]	1.8 (1.4-2.4)	2.3 (1.7-3.1)
Depression								
Unadjusted	0.6 (0.5-0.6)	1 [Reference]	2.1 (1.6-2.7)	2.5 (1.8-3.5)	0.4 (0.4-0.5)	1 [Reference]	2.4 (1.9-3.2)	2.9 (2.0-4.1)
Adjusted	0.6 (0.5-0.7)	1 [Reference]	2.0 (1.5-2.5)	2.3 (1.7-3.2)	0.5 (0.4-0.5)	1 [Reference]	2.3 (1.7-3.0)	2.5 (1.8-3.6)
Drowsiness								
Unadjusted	0.6 (0.5-0.7)	1 [Reference]	2.1 (1.6-2.6)	3.2 (2.4-4.2)	0.5 (0.4-0.6)	1 [Reference]	2.1 (1.7-2.8)	4.8 (3.6-6.3)
Adjusted	0.6 (0.5-0.7)	1 [Reference]	2.0 (1.6-2.5)	2.9 (2.2-3.9)	0.5 (0.4-0.6)	1 [Reference]	2.0 (1.6-2.6)	4.3 (3.3-5.7)
Appetite								
Unadjusted	0.6 (0.5-0.7)	1 [Reference]	2.4 (1.9-3.1)	2.7 (1.9-3.7)	0.5 (0.4-0.6)	1 [Reference]	3.1 (2.4-4.0)	3.6 (2.6-5.0)
Adjusted	0.6 (0.5-0.7)	1 [Reference]	2.3 (1.8-2.9)	2.5 (1.8-3.4)	0.5 (0.4-0.6)	1 [Reference]	2.9 (2.3-3.7)	3.2 (2.3-4.5)
Nausea								
Unadjusted	0.5 (0.5-0.6)	1 [Reference]	1.6 (1.1-2.3)	2.9 (1.9-4.4)	0.4 (0.4-0.5)	1 [Reference]	1.7 (1.1-2.5)	5.5 (3.9-7.8)
Adjusted	0.5 (0.5-0.6)	1 [Reference]	1.5 (1.1-2.1)	2.7 (1.8-4.0)	0.4 (0.4-0.5)	1 [Reference]	1.6 (1.1-2.3)	4.9 (3.5-7.0)
Pain								
Unadjusted	0.6 (0.5-0.7)	1 [Reference]	2.9 (2.3-3.7)	5.6 (4.2-7.4)	0.6 (0.5-0.7)	1 [Reference]	4.4 (3.4-5.6)	9.2 (7.0-12.1)
Adjusted	0.7 (0.6-0.8)	1 [Reference]	2.6 (2.1-3.3)	4.9 (3.7-6.5)	0.6 (0.5-0.7)	1 [Reference]	3.9 (3.0-5.0)	7.7 (5.8-10.2)
Dyspnea								
Unadjusted	0.5 (0.5-0.6)	1 [Reference]	1.7 (1.3-2.4)	2.8 (1.8-4.4)	0.4 (0.4-0.5)	1 [Reference]	2.3 (1.7-3.2)	5.3 (3.6-7.9)
Adjusted	0.5 (0.5-0.6)	1 [Reference]	1.7 (1.3-2.3)	2.9 (1.8-4.5)	0.4 (0.4-0.5)	1 [Reference]	2.3 (1.7-3.2)	5.4 (3.7-8.1)
Tiredness								
Unadjusted	0.7 (0.6-0.8)	1 [Reference]	2.0 (1.6-2.5)	2.8 (2.2-3.6)	0.5 (0.4-0.6)	1 [Reference]	1.9 (1.5-2.5)	3.8 (2.9-4.9)
Adjusted	0.7 (0.6-0.8)	1 [Reference]	2.0 (1.6-2.4)	2.6 (2.0-3.4)	0.5 (0.5-0.6)	1 [Reference]	1.9 (1.5-2.4)	3.5 (2.7-4.6)
Well-being								
Unadjusted	0.6 (0.5-0.7)	1 [Reference]	2.1 (1.7-2.5)	3.1 (2.3-4.1)	0.5 (0.4-0.6)	1 [Reference]	2.3 (1.8-2.9)	4.7 (3.5-6.2)
Adjusted	0.7 (0.6-0.8)	1 [Reference]	1.9 (1.6-2.4)	2.8 (2.1-3.7)	0.5 (0.5-0.6)	1 [Reference]	2.2 (1.7-2.7)	4.2 (3.2-5.6)

Abbreviation: HR, hazard ratio.

^a^ Adjusted values represent adjustment for age, sex, cancer type, time period, region, neighborhood income quintile, and rurality.

### Association of PC Involvement With Subsequent Symptom Improvement

Among the study cohort, 721 AYAs (13.3%) died within 5 years of their initial cancer diagnosis (eTable 2 in Supplement 1). Of these, 652 (90.4%) had at least 1 GPC visit; the median number of ESAS measurements was 5 (IQR, 2-11) before the first GPC visit and 6 (IQR, 2-15) afterward. A total of 612 patients (84.9%) had at least 1 SPC visit, with a median of 7 ESAS measurements (IQR, 3-13) before the first SPC visit and 5 (IQR, 2-13) afterward. When looking at AYAs who received GPC, 212 met our case definition and could be matched by sex and cancer type to at least 1 control patient. When examining AYAs who received SPC, 202 case-control pairs were identified. Characteristics of both sets of case-control pairs are shown in eTable 3 in Supplement 1.

eTable 4 in Supplement 1 presents the mean ESAS scores before and after the first GPC visit for case patients, corresponding scores for control patients, pre-to-post differences, and results of the multivariable linear regression model. Full model results are shown in eTable 5 in Supplement 1. Preindex scores were generally higher in case patients than in control patients. Improvement in scores was seen more often among case patients (who received GPC) than among control patients (who did not receive GPC). However, this difference between case and control patients was only statistically significant when examining pain scores. Among case patients, the mean pain ESAS score decreased from 3.40 to 2.95 after receiving GPC, while among control patients, mean pain score increased from 1.44 to 1.73 in the absence of GCP; this difference in trajectory was statistically significant, as indicated by the corresponding interaction term (*P* < .001). No such association between GPC and the trajectory of any other symptom was observed. Similar results were seen when examining the effectiveness of SPC (Table 3 and eTable 6 in Supplement 1). Sensitivity analyses of maximum ESAS scores vs mean ESAS scores yielded similar results.

**Table 3.** Preindex and Postindex Mean Edmonton Symptom Assessment System Scores Among Case and Control Patients (Specialty Palliative Care Analyses With 202 Pairs)

Symptom	Case patients			Control patients			Difference-in-differences (case-control)^b^	*P* value^c^
	Preindex mean score	Postindex mean score	Difference^a^	Preindex mean score	Postindex mean score	Difference^a^		
Anxiety	2.82	2.57	−0.25	2.15	2.03	−0.12	−0.13	.49
Depression	2.17	2.04	−0.12	1.61	1.54	−0.07	−0.05	.77
Drowsiness	2.94	3.08	0.13	1.90	1.91	0.02	0.12	.52
Appetite	2.70	2.58	−0.11	1.58	1.81	0.23	−0.34	.15
Nausea	1.61	1.56	−0.05	0.95	1.03	0.08	−0.13	.49
Pain	3.41	3.07	−0.34	1.86	2.16	0.30	−0.64	.003
Dyspnea	1.57	1.80	0.23	0.95	1.38	0.44	−0.20	.32
Tiredness	3.99	4.00	0.01	2.75	2.75	0.01	0.00	.97
Well-being	3.55	3.62	0.07	2.46	2.54	0.08	−0.01	.97

^a^ Negative values indicate mean postindex scores that were lower than mean preindex scores, whereas positive values indicate higher mean postindex scores. Higher values indicate increasing symptom severity.

^b^ Negative values indicate that case patients showed more improvement in scores compared with control patients, whereas positive values indicate that control patients showed more improvement. Higher values indicate increasing symptom severity.

^c^ *P* values correspond to statistical significance of the interaction term between case vs control patients and preindex vs postindex scores in multivariable linear regression, and thus whether the difference in change in preindex to postindex scores was significantly different between case and control patients.

## Discussion

In this population-based cohort study, we observed that AYAs reporting moderate or severe symptoms on ESAS screening were more likely to subsequently receive PC services compared with those reporting mild symptoms. Among decedents, both GPC and SPC involvement were associated with an improvement in reported pain severity, but they were not associated with improvement in other symptoms.

Adolescents and young adults have distinct developmental and psychosocial needs during cancer treatment and at the end of life (EOL).^11,27,28,29^ These patients have high EOL symptom burden, experience high-intensity care, and frequently die in the hospital.^13,30,31^ Despite endorsement of early PC integration,^9^ PC referrals are limited and occur late.^13,14^ Automatic triggers may increase the number and timeliness of PC referrals,^32,33,34^ including specific cancer-related events (metastatic disease, relapse). Our results suggest that patient-reported symptoms may serve a similar function, since in the context of a provincial symptom screening program, increasing symptom severity was associated with subsequent PC referral. We cannot determine whether such referrals were reliant on clinician initiative or triggered automatically. In addition, although analyses were adjusted for demographic factors and cancer type, we cannot rule out residual confounding by factors related to both symptom severity and PC involvement, such as cancer prognosis. Nonetheless, our findings support the possibility that routine screening leads to increased PC referrals, and that automatic triggers may further build on this success.

We and others have shown that PC involvement is associated with tangible benefits for AYAs, although most literature has focused on outcomes related to medical interventions near the EOL. For example, in a population-based Ontario decedent AYA cohort, PC involvement was associated with a 40% decrease in the odds of experiencing a composite measure of high-intensity EOL care and with a 65% decrease in the odds of mechanical ventilation at the EOL.^13^ Specialty PC involvement has been associated with decreased odds of high-intensity interventions, even compared with GPC.^35^

Although symptom control has been described as an essential component of PC and is a common reason for PC referrals,^9^ it is still unclear whether PC involvement reduces symptom severity. Two systematic reviews examined the association between PC and symptom control among adults with various conditions.^36,37^ Gaertner et al^36^ included 12 studies and found that although SPC had a small positive association with quality of life, no conclusions regarding symptom control were possible. A small association with pain control was noted, but only in studies of low methodologic quality and mainly driven by one study among adults with heart failure. Kavalieratos et al^37^ included 43 studies and transformed all symptom measures to ESAS scores. Although PC involvement was associated with a decrease in ESAS score of 1.0, the authors cautioned that methodologic rigor varied widely. When restricted to trials at low risk of bias, improvement in symptom score was only 0.3 and not statistically significant. Both reviews highlighted the difficulty of conducting randomized trials in this population and the need for clinical data.

The equivalent pediatric literature is similarly inconclusive. One systematic review included only 6 studies of symptom control; none used patient-reported measures and no clear association was noted.^38^ Two other studies looked at symptoms among pediatric patients before and after increased availability of PC services. In Germany, increased PC availability was associated with more symptom-directed treatment but with no difference in symptom presence or severity; the authors concluded that their symptom-directed treatment had not been successful.^39^ In a single-institution US study, there was no difference in presence of symptoms at the EOL, but there were fewer reports of suffering due to pain and dyspnea.^40^ Very few studies have examined this question specifically among AYAs. In one single-center publication, investigators compared AYAs with cancer who died with vs without PC involvement and found no difference in the number of symptoms.^41^

Using a difference-in-differences approach,^26^ we showed that in a larger and population-based AYA decedent cohort, both GPC and SPC involvement were associated with a small but statistically and clinically significant^37,42^ decrease in reported pain. However, no improvement in other symptoms was noted. Given the priority that AYA patients with cancer place on symptom control,^10^ this is a concern. Several explanations are possible. First, evidence-based guidelines exist for cancer pain management that detail opiate, nonopiate, and nonpharmacologic interventions.^43^ Involving PC professionals experienced in the implementation and personalization of these guidelines likely results in better pain management. The lack of similarly efficacious options for other symptoms, such as fatigue, dyspnea, and depression, may limit the effectiveness of even highly trained PC teams. Second, late involvement of PC may limit effective symptom control, particularly for symptoms slow to respond to interventions. In one randomized trial of PC intervention, its effectiveness on symptoms was not seen until 4 months had passed.^44^ Third, the degree of symptom control possible in the face of progressive symptoms at the EOL may be limited. Regardless, research priorities going forward must include the identification of efficacious interventions for nonpain physical and mental symptoms at the EOL that specifically account for the unique needs of AYAs.

### Strengths and Limitations

Study strengths include the large sample size, a focus on AYAs, population-based data, and the availability of patient-reported symptom scores. The difference-in-differences approach allowed for analysis of PC effectiveness while controlling for natural symptom trajectories.

Several limitations also merit consideration. First, ESAS screening occurs in the outpatient setting. Changes in symptom trajectory that occurred exclusively during hospitalization or at home would not have been captured. Second, although our difference-in-differences analysis was matched by age, sex, and cancer type, residual confounding is possible. Third, race and ethnicity variables were not available in our data sets, preventing examination of whether PC effectiveness varied by these characteristics. Fourth, our analysis cannot be generalized to AYAs treated in pediatric settings, because Ontario pediatric cancer centers do not use ESAS screening. Fifth, although the ESAS has been widely used for patients with cancer, including AYAs, it was not developed specifically for the AYA population. Patient-reported outcome measures specific to AYAs may have yielded different results. Finally, PC services provided by nonphysicians were not captured.

## Conclusions

In this cohort study of AYAs with cancer, those reporting moderate or severe symptoms in the context of a provincial screening program were more likely to subsequently receive PC services, suggesting that such programs have a role in ensuring PC involvement for AYAs in need. Although PC involvement was associated with a subsequent decrease in pain severity, no association with other symptoms was noted. New and more effective interventions targeting these other symptoms during cancer treatment and particularly at the EOL are urgently needed.

## Notes

Supplement 1. **eTable 1.** Population-Based Health Services Databases Used in This Study **eTable 2.** Demographic and Disease Characteristics of Decedent Subcohort at Time of Original Cancer Diagnosis (N = 721) **eTable 3.** Characteristics of Case-Control Pairs Based on General Palliative Care (n = 212) and Specialty Palliative Care (n = 202) **eTable 4.** Mean Preindex and Postindex ESAS Scores Among Case and Control Patients (General Palliative Care Analyses; n = 212 Pairs) for Each Symptom **eTable 5.** Full Linear Regression Model of Mean ESAS Scores Among Case and Control Patients (General Palliative Care Analyses) **eTable 6.** Full Linear Regression Model of Mean ESAS Scores Among Case and Control Patients (Specialty Palliative Care Analyses) **eAppendix 1.** Billing Codes Used to Define Palliative Care Visits **eAppendix 2.** Details of Cohort Creation **eFigure.** Flow Chart

Supplement 2.
Data Sharing Statement
