# Supplementary material for: Specialty Palliative Care and Symptom Severity and Control in Adolescents and Young Adults With Cancer
Source: JAMA Netw Open. 2023 Oct 20;6(10):e2338699. doi: 10.1001/jamanetworkopen.2023.38699 (PMC10589816; doi:10.1001/jamanetworkopen.2023.38699)
Supplement: Supplement 1. — eTable 1. Population-Based Health Services Databases Used in This Study eTable 2. Demographic and Disease Characteristics of Decedent Subcohort at Time of Original Cancer Diagnosis (N = 721) eTable 3. Characteristics of Case-Control Pairs Based on General Palliative Care (n = 212) and Specialty Palliative Care (n = 202) eTable 4. Mean Preindex and Postindex ESAS Scores Among Case and Control Patients (General Palliative Care Analyses; n = 212 Pairs) for Each Symptom eTable 5. Full Linear Regression Model of Mean ESAS Scores Among Case and Control Patients (General Palliative Care Analyses) eTable 6. Full Linear Regression Model of Mean ESAS Scores Among Case and Control Patients (Specialty Palliative Care Analyses) eAppendix 1. Billing Codes Used to Define Palliative Care Visits eAppendix 2. Details of Cohort Creation eFigure. Flow Chart [file jamanetwopen-e2338699-s001.pdf]

Supplementary Online Content

Gupta S, Li Q, Kassam A, et al. Specialty palliative care and symptom severity and control in adolescents and young adults with cancer. *JAMA Netw Open*. 2023;6(10):e2338699. doi:10.1001/jamanetworkopen.2023.38699

eTable 1. Population-Based Health Services Databases Used in This Study

eTable 2. Demographic and Disease Characteristics of Decedent Subcohort at Time of Original Cancer Diagnosis (N = 721)

eTable 3. Characteristics of Case-Control Pairs Based on General Palliative Care (n = 212) and Specialty Palliative Care (n = 202)

eTable 4. Mean Preindex and Postindex ESAS Scores Among Case and Control Patients (General Palliative Care Analyses; n = 212 Pairs) for Each Symptom

eTable 5. Full Linear Regression Model of Mean ESAS Scores Among Case and Control Patients (General Palliative Care Analyses)

eTable 6. Full Linear Regression Model of Mean ESAS Scores Among Case and Control Patients (Specialty Palliative Care Analyses)

eAppendix 1. Billing Codes Used to Define Palliative Care Visits

eAppendix 2. Details of Cohort Creation

eFigure. Flow Chart

This supplementary material has been provided by the authors to give readers additional information about their work.

© 2023 Gupta S et al. *JAMA Network Open*.

eTable 1. Population-Based Health Services Databases Used in This Study

Database	Data Elements	Description	Initiation Year
ALR	Cancer system therapies	Patient-level activity within the cancer system, focused on radiation therapy and systemic therapies, including chemotherapy. Also captures outpatient oncology visits	2005
DAD	Inpatient hospitalizations	One record per hospital admission including chart-abstracted demographic, clinical and outcome data	1988
NACRS/SDS	ED visits/Same day surgery	Demographic, clinical and disposition data	2000
OCR	Cancer diagnoses	One record per new cancer diagnosis	1964
OHIP	Physician claims	Claims for services billed by fee-for-service Ontario physicians. Physicians under alternative funding plans are also required to submit shadow claims, ensuring capture of nearly all physician encounters	1991
SMRD	Symptom scores	Patient-reported symptom burden	2007

ALR – Activity Level Reporting; DAD – Discharge Abstract Database; ED – emergency department; NACRS – National Ambulatory Care Reporting System; OCR – Ontario Cancer Registry; OHIP – Ontario Health Insurance Plan Claims Database; SMRD – Symptom Management Reporting Database; SDS – Same Day Surgery

© 2023 Gupta S et al. *JAMA Network Open*.

eTable 2. Demographic and Disease Characteristics of Decedent Subcohort at Time of Original Cancer Diagnosis (N = 721)

Characteristic	**N (%)**
Age (years) [Median (IQR)]	25 (21-27)
Sex	
Male	364 (50.5)
Female	357 (49.5)
Time period	
Early (2010-2014)	434 (60.2)
Late (2015-2018)	287 (39.8)
Neighborhood income quintile	
Rural	91 (12.6)
Urban Q1 (lowest)	138 (19.1)
Urban Q2	125 (17.3)
Urban Q3	125 (17.3)
Urban Q4	123 (17.1)
Urban Q5 (highest)	119 (16.5)
Cancer type	
Hematologic	186 (25.8)
Solid tumor	442 (61.3)
CNS	93 (12.9)
Region	
Central	211 (29.3)
East	177 (24.5)
North	59 (8.2)
Toronto	58 (8.0)
West	216 (30.3)

CNS – central nervous system; IQR – interquartile range; N – number

© 2023 Gupta S et al. *JAMA Network Open*.

eTable 3. Characteristics of Case-Control Pairs Based on General Palliative Care (n = 212) and Specialty Palliative Care (n = 202)

Characteristic	General Palliative Care			Specialty Palliative Care		
	Cases	Controls	p-value	Cases	Controls	p-value
Age [years; median (IQR)]	25 (22-27)	25 (22-28)	0.50	25 (22-27)	25 (22-28)	0.53
Sex [N (%)]			1.00			1.00
Male	98 (46.2)	98 (46.2)		90 (44.6)	90 (44.6)	
Female	114 (53.8)	114 (53.8)		112 (55.4)	112 (55.4)	
Time period [N (%)]			0.91			0.84
Early (2010-2014)	136 (64.2)	137 (64.6)		129 (63.9)	127 (62.9)	
Late (2015-2018)	76 (35.8)	75 (35.4)		73 (36.1)	75 (37.1)	
Neighborhood income quintile [N (%)]			0.84			0.10
Rural	22 (10.4)	18 (8.5)		19 (9.4)	27 (13.4)	
Urban Q1 (lowest)	43 (20.3)	42 (19.8)		40 (19.8)	35 (17.3)	
Urban Q2	32 (15.1)	35 (16.5)		31 (15.3)	46 (22.8)	
Urban Q3	47 (22.2)	39 (18.4)		44 (21.8)	26 (12.9)	
Urban Q4	39 (18.4)	43 (20.3)		38 (18.8)	39 (19.3)	
Urban Q5 (highest)	29 (13.7)	35 (16.5)		30 (14.9)	29 (14.4)	
Cancer type [N (%)]			0.29			0.34
Hematologic	31 (14.6)	31 (14.6)		29 (14.4)	29 (14.4)	
Melanoma	11 (5.2)	23 (10.8)		9 (4.5)	18 (8.9)	
CNS	21 (9.9)	21 (9.9)		20 (9.9)	20 (9.9)	
Sarcoma	30 (14.2)	21 (9.9)		25 (12.4)	19 (9.4)	
Testicular/Ovarian	17 (8.0)	18 (8.5)		17 (8.4)	28 (13.9)	
Breast	28 (13.2)	32 (15.1)		26 (12.9)	26 (12.9)	
Colorectal	11 (5.2)	16 (7.5)		12 (5.9)	9 (4.5)	
Other	63 (29.7)	50 (23.6)		64 (31.7)	53 (26.2)	
Region [N (%)]			0.01			0.21
Central	60 (28.3)	57 (26.9)		57 (28.2)	72 (35.6)	
East	62 (29.2)	51 (24.1)		59 (29.2)	44 (21.8)	
North	17 (8.0)	10 (4.7)		18 (8.9)	12 (5.9)	
Toronto	16 (7.5)	7 (3.3)		17 (8.4)	15 (7.4)	
West	57 (26.9)	87 (41.0)		51 (25.2)	59 (29.2)	
N of ESAS scores pre-index [median (IQR)]	3 (1-4)	3 (2-4)	0.48	3 (2-4)	3 (2-4)	0.98
N of ESAS scores post-index [median (IQR)]	3 (2-4)	2 (1-4)	0.03	3 (2-4)	2 (1-4)	0.01

CNS – central nervous system; IQR – interquartile range; N – number

© 2023 Gupta S et al. *JAMA Network Open*.

eTable 4. Mean Preindex and Postindex ESAS Scores Among Case and Control Patients (General Palliative Care Analyses; n = 212 Pairs) for Each Symptom

Symptom	Cases			Controls			Difference-in-Difference (Case-Control)[b]	p-value[c]
	Mean Pre-Score	Mean Post-Score	Post-Pre Difference[a]	Mean Pre-Score	Mean Post-Score	Post-Pre Difference[a]		
Anxiety	2.84	2.63	-0.21	1.79	1.85	0.05	-0.26	0.15
Depression	2.18	2.14	-0.04	1.24	1.35	0.12	-0.16	0.32
Drowsiness	2.88	3.01	0.14	1.70	1.74	0.04	0.10	0.58
Appetite	2.60	2.60	0.00	1.48	1.58	0.10	-0.10	0.63
Nausea	1.60	1.59	-0.01	0.89	0.84	-0.05	0.04	0.84
Pain	3.40	2.95	-0.45	1.44	1.73	0.29	-0.74	<0.001
Dyspnea	1.48	1.67	0.19	0.67	0.77	0.10	0.09	0.57
Tiredness	4.03	3.97	-0.06	2.44	2.54	0.10	-0.16	0.42
Wellbeing	3.56	3.53	-0.03	2.08	2.23	0.14	-0.18	0.32

[a]Negative values (shaded in blue) indicate mean post-scores that were lower than mean pre-scores, while positive values (shaded in red) indicate higher mean post-scores. Higher values indicate increasing symptom severity.

[b]Negative values (shaded in green) indicate that cases showed more improvement in scores compared to controls, while positive values (shaded in yellow) indicate that controls showed more improvement.

[c]P-values correspond to statistical significance of the interaction term between case vs. control and pre vs. post in multivariable linear regression, and thus whether the difference in change in pre-post scores statistically significantly different between cases and controls.

© 2023 Gupta S et al. *JAMA Network Open*.

eTable 5. Full Linear Regression Model of Mean ESAS Scores Among Case and Control Patients (General Palliative Care Analyses)

Symptom	Parameter	Estimate	Standard Error	P-value
Anxiety	Intercept	1.7918	0.1495	<0.001
	Case vs. control	1.0525	0.2438	<0.001
	Pre vs. Post	0.0542	0.1201	0.65
	(CaseControl)*(PrePost)	-0.2648	0.1819	0.15
Depression	Intercept	1.2376	0.1228	<0.001
	Case vs. control	0.9414	0.2126	<0.001
	Pre vs. Post	0.1155	0.0855	0.18
	(CaseControl)*(PrePost)	-0.1556	0.1549	0.32
Drowsiness	Intercept	1.7039	0.1401	<0.001
	Case vs. control	1.1732	0.2261	<0.001
	Pre vs. Post	0.0405	0.0974	0.68
	(CaseControl)*(PrePost)	0.0960	0.1752	0.58
Appetite	Intercept	1.4769	0.1339	<0.001
	Case vs. control	1.1218	0.2151	<0.001
	Pre vs. Post	0.0997	0.1217	0.41
	(CaseControl)*(PrePost)	-0.1004	0.2067	0.63
Nausea	Intercept	0.8898	0.0966	<0.001
	Case vs. control	0.7062	0.1721	<0.001
	Pre vs. Post	-0.0451	0.1079	0.68
	(CaseControl)*(PrePost)	0.0364	0.1760	0.84
Pain	Intercept	1.4373	0.1172	<0.001
	Case vs. control	1.9675	0.2215	<0.001
	Pre vs. Post	0.2916	0.1323	0.03
	(CaseControl)*(PrePost)	-0.7442	0.2026	<0.001
Dyspnea	Intercept	0.6667	0.0835	<0.001
	Case vs. control	0.8171	0.1640	<0.001
	Pre vs. Post	0.1012	0.0829	0.22
	(CaseControl)*(PrePost)	0.0958	0.1669	0.57
Tiredness	Intercept	2.4428	0.1468	<0.001
	Case vs. control	1.5903	0.2374	<0.001
	Pre vs. Post	0.0994	0.1266	0.43
	(CaseControl)*(PrePost)	-0.1597	0.1964	0.41
Wellbeing	Intercept	2.0826	0.1375	<0.001
	Case vs. control	1.4769	0.2182	<0.001
	Pre vs. Post	0.1430	0.1055	0.18
	(CaseControl)*(PrePost)	-0.1759	0.1774	0.32

© 2023 Gupta S et al. *JAMA Network Open*.

eTable 6. Full Linear Regression Model of Mean ESAS Scores Among Case and Control Patients (Specialty Palliative Care Analyses)

Symptom	Parameter	Estimate	Standard Error	P-value
Anxiety	Intercept	2.1520	0.1777	<0.001
	Case vs. control	0.6659	0.2616	0.01
	Pre vs. Post	-0.1195	0.1212	0.32
	(CaseControl)*(PrePost)	-0.1333	0.1913	0.49
Depression	Intercept	1.6074	0.1656	<0.001
	Case vs. control	0.5580	0.2371	0.02
	Pre vs. Post	-0.0709	0.1127	0.53
	(CaseControl)*(PrePost)	-0.0498	0.1702	0.77
Drowsiness	Intercept	1.8978	0.1519	<0.001
	Case vs. control	1.0466	0.2400	<0.001
	Pre vs. Post	0.0167	0.1094	0.88
	(CaseControl)*(PrePost)	0.1180	0.1850	0.52
Appetite	Intercept	1.5767	0.1404	<0.001
	Case vs. control	1.1188	0.2181	<0.001
	Pre vs. Post	0.2299	0.1634	0.16
	(CaseControl)*(PrePost)	-0.3404	0.2349	0.15
Nausea	Intercept	0.9492	0.1146	<0.001
	Case vs. control	0.6602	0.1825	<0.001
	Pre vs. Post	0.0813	0.1283	0.53
	(CaseControl)*(PrePost)	-0.1326	0.1899	0.49
Pain	Intercept	1.8610	0.1518	<0.001
	Case vs. control	1.5509	0.2419	<0.001
	Pre vs. Post	0.3017	0.1452	0.04
	(CaseControl)*(PrePost)	-0.6395	0.2127	0.002
Dyspnea	Intercept	0.9527	0.1104	<0.001
	Case vs. control	0.6161	0.1786	<0.001
	Pre vs. Post	0.4271	0.1120	<0.001
	(CaseControl)*(PrePost)	-0.1835	0.1852	0.32
Tiredness	Intercept	2.7471	0.1534	<0.001
	Case vs. control	1.2398	0.2421	<0.001
	Pre vs. Post	0.0051	0.1310	0.97
	(CaseControl)*(PrePost)	0.0070	0.1949	0.97
Wellbeing	Intercept	2.4580	0.1476	<0.001
	Case vs. control	1.0910	0.2277	<0.001
	Pre vs. Post	0.0824	0.1297	0.53
	(CaseControl)*(PrePost)	-0.0074	0.1987	0.97

© 2023 Gupta S et al. *JAMA Network Open*.

eAppendix 1. Billing Codes Used to Define Palliative Care Visits

A901 GP/FP house call (billed with B997 or B998)
A902 Pronouncement of death in home (billed with B997 or B998)
A945 GP/FP special palliative care consultation
B966 Travel premium-palliative care home visit
B990 Special visit to patient's home, weekday/daytime
B992 Special visit to patient's home, weekday/daytime, sacrifice office hours
B994 Special visit to patient's home, non-elective, evening hours
B996 Special visit to patient's home, night time (first patient)
B997 Special visit to patient's home, palliative care, days, evenings (from 2009)
B998 Special visit to patient's home, palliative care, days, evenings (from 2005)
C882 GP/FP terminal care in hospital
C982 Palliative care, hospital in patient
C945 Special palliative care consultation, hospital in patient
E083 Subsequent visit as most responsible physician (billed with C882 or C982 or C945)
K015 Counseling a relative on behalf of a patient
K023 Palliative care support to individual, 30 min
K700 Palliative Care Out-patient Case Conference
W872 Terminal care in nursing home, GP/FP practice
W882 Terminal care in chronic care hospital, GP/FP
G511 Telephone management of palliative care at home
G512 Weekly palliative care case management

© 2023 Gupta S et al. *JAMA Network Open*.

eAppendix 2. Details of Cohort Creation

Of 9,399 identified patients, 649 (6.9%) were diagnosed and treated at pediatric institutions. Of the 8,750 AYA diagnosed and treated at adult institutions, 2,949 (33.7%) had no cancer-related visit within the first year of diagnosis, as indicated by the Activity Level Reporting (ALR) database, and were thus excluded. The ALR database includes data on patient level cancer-related activity (e.g. radiation and systemic therapies, outpatient oncology clinic visits). Confirming previous assumptions that such patients represent cancer diagnoses that do not require services at a cancer center, 1,416 (48.0%) had thyroid cancer, only 47 (0.02%) died within a year of diagnosis, and only 82 (0.03%) were ESAS screened within a year. Of the remaining 5,801 patients with ALR visits within the first year, 366 (6.3%) only had ALR visits at non-RCCs and thus also excluded. These excluded patients were less likely to live in rural areas [13/366 (3.6%) vs. 535/5435 (9.8%); p<0.001] and more likely to be diagnosed in the later time period [242/366 (66.1%) vs. 2,175/5,435 (40.0%); p<0.001].

© 2023 Gupta S et al. *JAMA Network Open*.

eFigure. Flow Chart

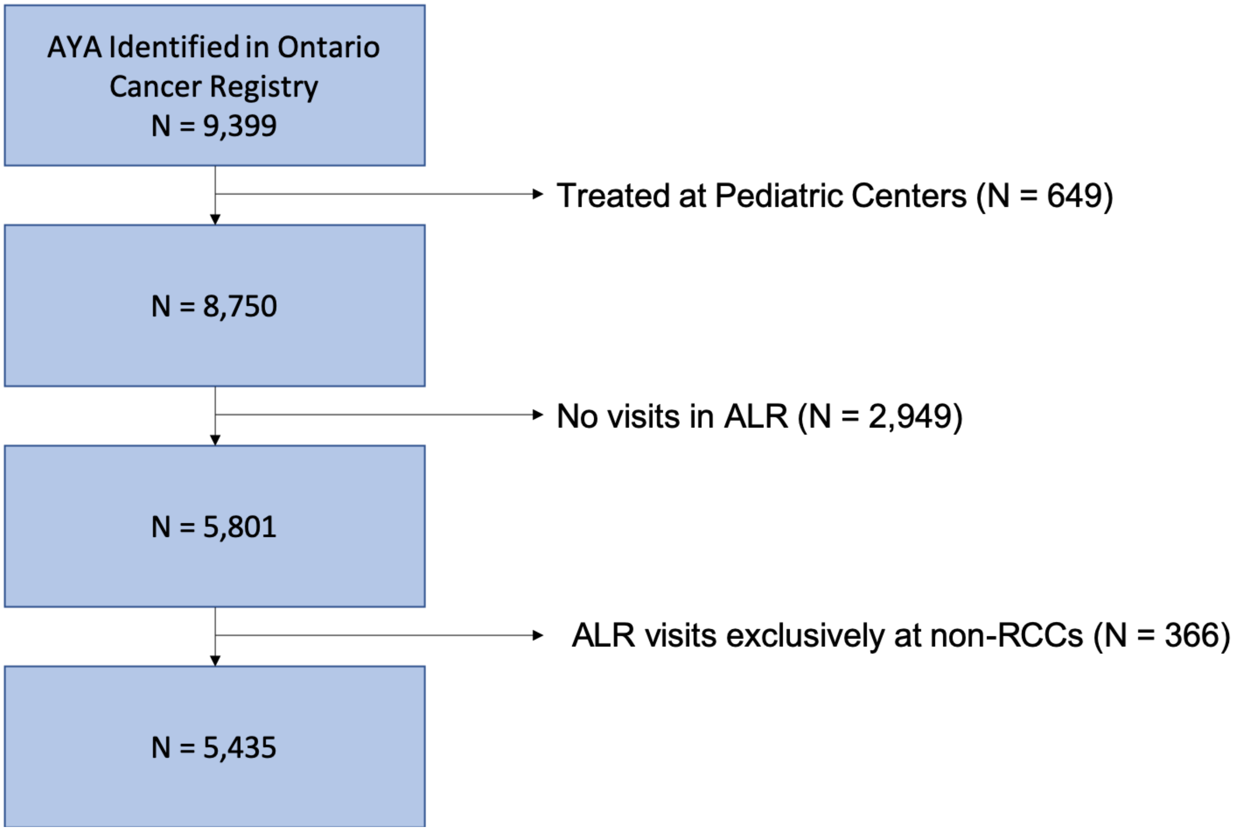

ALR – Activity Level Reporting database; AYA – Adolescent and young adult; RCC – Regional cancer center

© 2023 Gupta S et al. *JAMA Network Open*.
