# Supplementary material for: Specialty Palliative Care and Symptom Severity and Control in Adolescents and Young Adults With Cancer
Source: JAMA Netw Open. 2023 Oct 20;6(10):e2338699. doi: 10.1001/jamanetworkopen.2023.38699 (PMC10589816; doi:10.1001/jamanetworkopen.2023.38699)
Supplement: Supplement 2. — Data Sharing Statement [file jamanetwopen-e2338699-s002.pdf]

Data Sharing Statement

Gupta. Specialty Palliative Care and Symptom Severity and Control in Adolescents and Young Adults With Cancer. *JAMA Netw Open*. Published October 20, 2023. doi:10.1001/jamanetworkopen.2023.38699

Data
Data available: No

Additional Information
Explanation for why data not available: The data involve personal health information; Ontario privacy legislation presents the disclosure of such information
